# Systemic Inflammation and Growth in Children Born to Mothers With and Without HIV in Rural Zimbabwe

**DOI:** 10.1093/ofid/ofaf810

**Published:** 2026-01-05

**Authors:** Ceri Evans, Jonathan P Sturgeon, Sandra Rukobo, Margaret Govha, Bernard Chasekwa, Florence D Majo, Batsirai Mutasa, Naume Tavengwa, Robert Ntozini, Jean H Humphrey, Kuda Mutasa, Andrew J Prendergast

**Affiliations:** Zvitambo Institute for Maternal and Child Health Research, Harare, Zimbabwe; Department of Clinical Infection, Microbiology and Immunology, University of Liverpool, Liverpool, UK; Blizard Institute, Queen Mary University of London, London, UK; Zvitambo Institute for Maternal and Child Health Research, Harare, Zimbabwe; Blizard Institute, Queen Mary University of London, London, UK; Section of Paediatrics, Imperial College London, London, UK; Zvitambo Institute for Maternal and Child Health Research, Harare, Zimbabwe; Zvitambo Institute for Maternal and Child Health Research, Harare, Zimbabwe; Zvitambo Institute for Maternal and Child Health Research, Harare, Zimbabwe; Zvitambo Institute for Maternal and Child Health Research, Harare, Zimbabwe; Zvitambo Institute for Maternal and Child Health Research, Harare, Zimbabwe; Zvitambo Institute for Maternal and Child Health Research, Harare, Zimbabwe; Zvitambo Institute for Maternal and Child Health Research, Harare, Zimbabwe; Zvitambo Institute for Maternal and Child Health Research, Harare, Zimbabwe; Department of International Health, Johns Hopkins Bloomberg School of Public Health, Baltimore, Maryland, USA; Zvitambo Institute for Maternal and Child Health Research, Harare, Zimbabwe; Zvitambo Institute for Maternal and Child Health Research, Harare, Zimbabwe; Blizard Institute, Queen Mary University of London, London, UK

**Keywords:** children, growth, HIV-exposed, inflammation, vascular inflammation

## Abstract

**Background:**

Sixteen million children are HIV-exposed but uninfected (HEU) due to the prevention of vertical transmission. Despite avoiding HIV, children who are HEU face higher risks of infections and poorer growth and development than children HIV-unexposed (HU), though mechanisms remain unclear. We hypothesized that systemic and vascular inflammations contribute to disparities.

**Methods:**

The Sanitation Hygiene Infant Nutrition Efficacy (SHINE) trial recruited pregnant women at ∼12 gestational weeks between 2012 and 2015 in rural Zimbabwe (∼15% HIV prevalence, >80% antiretroviral therapy coverage). Plasma biomarkers were measured using enzyme-linked immunosorbent assay (ELISA) and multiplex assays in a subgroup of children at 1 month of age and compared using generalized estimating equations adjusted for trial arm, maternal age, birthweight, prematurity, sex, and age. Principal component analysis was used to reduce dimensionality of biomarkers.

**Results:**

Seventy-one children who are HEU and 62 who are HU were included. Twenty-two of 27 biomarkers were raised in HEU versus HU. Systemic inflammatory markers (IL-1β/interferon-γ/TNF-α/sCD14) and vascular activation markers (L-selectin/VCAM-1) were significantly higher. HIV-exposed but uninfected infants gained 6.1 g/day less than HU infants in the first month after birth. Although one principal component, primarily driven by vascular endothelial growth factor, was associated with increased growth rate, the difference between HEU and HU growth trajectories was not affected by differences in any principal components, suggesting that inflammation does not explain lower growth amongst HEU children.

**Conclusions:**

Children who are HEU have significantly elevated systemic and vascular inflammatory biomarkers compared with those who are HU. Understanding causes and consequences of this inflammatory imbalance may identify new intervention targets for improving outcomes in this vulnerable group.

Globally, there are over 16 million children who were exposed to HIV (HIV-1) during pregnancy but remain uninfected due to the success of prevention of vertical HIV transmission programs [1]. Despite avoiding infection, children who are HIV-exposed but uninfected (HEU) have a higher risk of infectious morbidity and mortality and poorer growth and neurodevelopment compared with children who are HIV-unexposed (HU), but the underlying mechanisms are uncertain [2–5].

We recently explored health outcomes amongst children in the Sanitation Hygiene Infant Nutrition Efficacy (SHINE) trial in rural Zimbabwe, demonstrating a greater risk of mortality in children who are HIV-exposed compared with children who are HIV-unexposed [6]. We went on to explore potential biological pathways underlying infant mortality and found that maternal factors including HIV disease severity and coinfections shape immune development in children who are HEU [7]. We also showed that the maternal inflammatory milieu during pregnancy is related to infant mortality. We showed that children who are HEU and survived to 18 months had growth and neurodevelopmental impairments relative to children born to mothers without HIV, both in early life [6, 8] and later in childhood [9]. Collectively, these findings suggest that the “immune footprint” of HIV exposure has long-lasting effects across the life-course.

To progress our hypothesis that proinflammatory changes contribute to clinical disparities amongst children who are HEU, we set out to further characterize the inflammatory milieu in early life.

## METHODS

### Study Population

Participants were recruited to the SHINE trial, a 2 × 2 factorial cluster-randomized trial in rural Zimbabwe, which enrolled women in pregnancy between 22 November 2012 and 27 March 2015, and assessed the effects of improved infant and young child feeding (IYCF) and improved household water, sanitation, and hygiene (WASH) on stunting and anemia amongst their children at age 18 months. The design, methods, and primary results of the SHINE trial have been reported in detail previously [10–14]. The SHINE trial is registered at ClinicalTrials.gov (NCT01824940).

### Data Collection

Women were enrolled in early pregnancy, and research nurses collected baseline maternal data, anthropometry, and blood at median 14 gestational weeks. Women had another home visit at median 32 gestational weeks for repeat data and blood collection. Home visits were scheduled at 1, 3, 6, 12, and 18 months postpartum, with collection of biological specimens in a subgroup of children. At 1 month of age (or as soon as possible thereafter within the allowable visit window up to 90 days of age), blood was collected from children into endotoxin-free EDTA tubes (BD Biosciences).

### HIV Testing

Women and children were tested longitudinally for HIV, as previously described [6]. Children who are HEU were defined as infants born to women living with HIV during pregnancy but who themselves remained HIV-negative through 18 months of age [6]. HIV-unexposed children were born to women without HIV infection at delivery.

### Selection of Infants

HIV-exposed children who survived to 18 months were eligible for inclusion in an immunology substudy [7] if they had length measured at 18 months of age, laboratory samples available from the 1-month visit, and mothers with available laboratory samples from the pregnancy baseline visit (N = 143). One HU child meeting these criteria was chosen for each HEU child, matched on trial arm, sex, and season of birth. If more than 1 HU child met the matching criteria, 1 child was chosen at random, to provide a matched group of HIV-unexposed children. Children with plasma availability following completion of the immunology substudy were included in the current analysis (Supplementary Figure 1).

### Biomarker Assays

Infant plasma was analyzed using a custom 24-plex Luminex assay (R&D Systems, assay ref: B829YuzuZ) which included inflammatory cytokines (TNF-α, interleukin-6 [IL-6], IL-33, IL-1β, interferon-γ, IL-1ra, CCL3, CCL4, IL-8, IL-2, IL-10), markers of vascular/endothelial activation (D-dimer, L-selectin, thrombopoietin, intercellular adhesion molecule-1 [ICAM-1], vascular adhesion molecule-1 [VCAM-1], ICAM1, eotaxin), and growth factors (epidermal growth factor [EGF], vascular endothelial growth factor [VEGF], PlGF, angiopoietin-1, granulocyte colony-stimulating factor [GCSF], granulocyte–macrophage colony-stimulating factor [GM-CSF], insulin-like growth factor binding protein-3 [IGFBP-3]; Supplementary Table 1). Samples were run on a Luminex Magpix machine (Luminex Corp) in Zimbabwe, with roughly equal proportions of HEU samples on each plate and all plates run on the same day. Soluble CD14 (a marker of monocyte activation) and C-reactive protein (CRP) were measured in child plasma by enzyme-linked immunosorbent assay (ELISA) as markers of systemic and monocyte activation, respectively, using R&D Systems Quantikine kits according to the manufacturer's instructions. Soluble CD163 (a marker of monocyte activation) was measured in plasma by ELISA (R&D Systems DuoSet) according to the manufacturer's instructions. HIV viral loads were measured in 200 μL of plasma, using the Abbott RealTime HIV-1 extraction assay followed by real-time PCR on the Abbott m2000rt platform using the Abbott RealTime HIV Amplification Reagent Kit. The limit of detection of the assay was 150 copies/mL. For cytomegalovirus (CMV), viral nucleic acid was extracted from 100 μL of plasma using the QIAamp DSP Virus Spin Kit. Cytomegalovirus was detected by real-time PCR on the Abbott m2000rt platform using the Abbott RealTime CMV Amplification Reagent Kit modified for the nucleic acid extraction method used, providing a limit of quantification of 45 copies/mL. Laboratory scientists were blinded to HIV/CMV exposure status for all analyses.

### Statistical Analyses

We compared baseline characteristics between HEU and HU children while handling within-cluster correlation using multinomial and ordinal regression models with robust variance estimation and Somers’ D for medians. Generalized estimating equations with robust variance estimation and an exchangeable correlation structure to account for cluster were used to compare log_10_-normalized concentrations of biomarkers between groups, adjusted for randomized trial arm, maternal age, infant birthweight, prematurity, infant sex, and exact infant age at the 1-month study visit (for child samples). *P*-values were adjusted for multiple comparisons using the Benjamini–Hochberg procedure to control the false discovery rate (FDR), and adjusted *P*-values (*q*-values) were calculated. A principal component analysis (PCA) was conducted using log_10_-transformed biomarker variables, which were then zero centered and standardized. We explored the relationship between each component (with an eigenvalue of >1) and child weight gain over the first month after birth, calculated as the difference between weight at the 1-month visit and birthweight, divided by the exact number of days between the two measurements. These analyses used generalized estimating equation (GEE) models that explored the association between component score and child growth, including the same covariates as above. The impact of detectable maternal HIV or CMV viremia on the infant principal component score was assessed by testing for significance when adding in the indicator variable for HIV or CMV in the GEE model. Stata version 18.5 was used for all analyses.

### Patient Consent Statement

All mothers provided written informed consent to join the study. The Medical Research Council of Zimbabwe (MRCZ/A/1675) and Johns Hopkins Bloomberg School of Public Health (JHU IRB # 4205) approved the study protocol.

## RESULTS

Seventy-one HEU children and 62 HU children were included. Mothers living with HIV tended to be older than mothers without HIV (mean 30.3 years vs 27.2 years; *P* = .007), but baseline characteristics were otherwise similar between the two groups (Table 1).

**Table 1.** Baseline Characteristics of Children Included in This Study

Baseline Characteristics^a^	HIV-exposed	HIV-unexposed	*P*
Mothers (N)	69	60	
Infants (N)	71	62	
**Trial arm**	…	…	
SOC	10 (14.1%)	12 (19.4%)	.80
IYCF	24 (33.8%)	19 (30.7%)	
WASH	17 (23.9%)	15 (24.2%)	
IYCF + WASH	20 (28.2%)	16 (25.8%)	
**Household characteristics**	…	…	
Wealth quintile^b^	…	…	
Lowest	17 (24.6%)	10 (16.7%)	
Second	17 (24.6%)	17 (28.3%)	.17
Middle	20 (29.0%)	9 (15.0%)	
Fourth	7 (10.1%)	14 (23.3%)	
Highest	8 (11.6%)	10 (16.7%)	
**Maternal characteristics**	…	…	
Age, years; mean (SD)	30.3 (6.4)	27.2 (6.7)	.007
MUAC, cm; mean (SD)	26.2 (2.6)	27.2 (3.5)	.09
Completed schooling, years; mean (SD)	9.2 (2.1)	9.6 (1.5)	.20
Married	60 (90.9%)	56 (93.3%)	.63
Employed	5 (7.4%)	6 (10.0%)	.64
Parity; median (IQR)	3 (2, 3)	2 (1, 3)	.25
Maternal CD4 count^c^; mean (SD)	463 (244)	-	-
Co-trimoxazole prophylaxis during pregnancy^d^	51/69 (73.9%)	-	-
Maternal ART during pregnancy^e^	66/69 (95.7%)	-	-
Tenofovir-based ART regimen	59/66 (89.4%)	-	-
Zidovudine-based ART regimen	3/66 (4.5%)	-	-
Other/unknown regimen^f^	4/66 (6.1%)	-	-
Detectable HIV viral load at recruitment	30/63 (47.6%)	-	-
Detectable CMV viremia at recruitment	20/63 (31.7%)	10/58 (17.2%)	.03
Religion	…	…	
Apostolic	33 (47.8%)	25 (41.7%)	.80
Other Christian religions	31 (44.9%)	30 (50.0%)	
Other non-Christian religions	5 (7.3%)	5 (8.3%)	
**Infant characteristics**	…	…	
Female	32 (45.1%)	30 (48.4%)	.67
Birth weight, kg; mean (SD)	2.97 (0.53)	3.13 (0.54)	.08
Birth weight < 2500 g	10 (14.1%)	7 (11.3%)	.77
Premature	16 (22.5%)	11 (17.7%)	.49
Age (days) at time of blood test; median (IQR)	38 (35, 51)	45 (35, 59)	.07

Abbreviations: SD: standard deviation; IQR: interquartile range; MUAC: mid-upper arm circumference; SOC, standard of care; IYCF, infant and young child feeding; WASH, water and sanitation/hygiene.

^a^Baseline for mothers was 2 weeks after consent (∼14 weeks gestation). Baseline for infants was at birth. Values are %, unless stated.

^b^Wealth index constructed as described in Chasekwa et al [15].

^c^CD4 count at baseline visit or at 32 gestational week visit if no baseline result.

^d^Documented exposure to co-trimoxazole during pregnancy.

^e^Documented exposure to antiretroviral therapy during pregnancy.

^f^Includes nontenofovir- or zidovudine-based regimens, use of both tenofovir and zidovudine during pregnancy (including switching regimens), or undocumented antiretroviral therapy regimen.

### Systemic Inflammation

**Figure 1.**
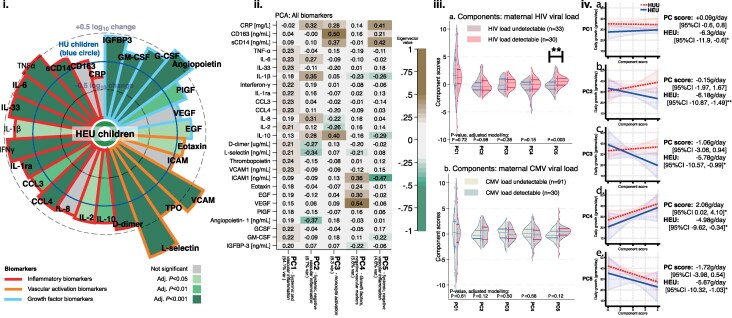
i) The level of biomarkers in HEU children compared with HU children; ii) the principal component analysis of the biomarkers; iii) association of component scores with maternal HIV and CMV viral load; iv) association of the component scores with observed growth, by HEU/HU status. i) Graph showing the adjusted^1^ log_10_ biomarker value in the HEU group compared with the HU group. The HU group level is shown at the size of the reference circular line. If the ray is outside this, then the adjusted log10 value in HEU children is above the HU children. If the ray is colored, this was significant following Benjamini–Hochberg correction for false discovery rate; ii) the principal component analysis results showing component score of each biomarker, which had been log_10_ normalized, zero centered, and standardized; iii) the impact of maternal detectable viral load at recruitment for a) HIV and b) CMV; iv) the outcome of adjusted modeling of association of each component score with linear growth, split by HEU/HU status. The *P*-value reporting the significance was following adjusted^1^ analysis. ^1^Adjustments for all adjusted analyses were age in days, sex, trial arm, birthweight, maternal age, and prematurity. Where significance is indicated by stars: *P < .05; **P < .01; ***P < .001. HEU, HIV-exposed uninfected children; HU, HIV-unexposed.

Most inflammatory biomarkers were significantly higher in HEU compared with HU children (Figure 1i; Supplementary Table 2). The most pronounced differences were for L-selectin (adjusted log_10_ difference +1.05 ng/mL, 95% CI .69, 1.42, *P* < .001), VCAM-1 (+0.73 ng/mL, 95% CI .43, 1.04, *P* < .001), and angiopoietin (+0.65 ng/mL, 95% CI .33, .98*, P* < .001), which are endothelial activation markers and growth factor markers reflecting vascular inflammation, followed by the systemic inflammatory biomarker TNF-α (adjusted log_10_ difference 0.52 pg/mL, 95% CI .33, .72, *P* < .001), which has broad proinflammatory actions.

A PCA was carried out to reduce data dimensionality, with 5 components having an eigenvalue > 1. Component 1 explained over half (54.1%) of the variance in the biomarkers, containing 14 analytes with a loading of >0.20, which reflected systemic inflammation, vascular inflammation, and growth factors (Figure 1ii). Component 2 represented systemic inflammation with negative vascular inflammatory biomarkers; component 3 had biomarkers that likely represented monocyte activation; component 4 comprised growth factors and negative vascular biomarkers; and component 5 reflected systemic inflammatory markers, with negative vascular markers.

Examining the effect of maternal HIV viral load at recruitment, we compared infants whose mothers had undetectable versus detectable viral load. The two groups did not show significantly different scores for components 1–4, but detectable HIV viral load was associated with an increased level of component 5, a component primarily composed of CRP, sCD14, and negative ICAM-1 (Figure 1iii-a). Both groups were included when examining CMV viremia: detectable maternal CMV viremia at recruitment did not significantly contribute to the levels of the scores for any component (Figure 1iii-b).

### Growth

The unadjusted rate of growth was 29.7 g/day in HEU infants and 34.1 g per day in HU infants between birth and the 1-month visit. We conducted an adjusted analysis to compare HEU growth using GEE modeling with weight gain as the dependent variable and including birthweight, prematurity, sex, trial arm, maternal age, and age in days as covariates, finding that HEU children gained 6.1 g (95% CI 1.6, 10.6) less per day compared with HU children. Next, we explored whether inflammatory pathways may underlie this difference by adding each principal component into the adjusted model. Component 4, which included the growth factors VEGF and EGF, as well as ICAM-1 and negative L-selectin and negative IL-1β, was independently associated with increased growth (2.06 g/day, 95% CI .02, 4.10; Figure 1iv-d), and component 5, which included the systemic inflammatory markers CRP, sCD14, and sCD163, as well as negative IL-1β, negative IL-10, and negative ICAM-1, had a trend toward an independent association with reduced growth (−1.72 g/day, 95% CI −3.98, .54) (Figure 1iv-e). However, individually adding these principal component scores to the HEU growth model did not alter the HEU effect size or its significance (Figure 1iv-d,e). Other components did not have independent or associations with growth and did not alter the HEU effect size or its significance (Figure 1iv-a,b,c).

## DISCUSSION

In this substudy of rural Zimbabwean children in the SHINE trial, we found that inflammatory biomarkers were significantly raised in children who are HEU compared with children who are HU in early infancy. Elevation of plasma TNF-α, IL-1β, CCL3, CCL4, and IL-10 suggests that a broad proinflammatory and immunoregulatory response characterizes early life in infants who are HEU. Elevated sCD14 and sCD163 indicate monocyte activation, while high L-selectin and VCAM-1 suggest vascular inflammation. This work builds upon our previous findings of elevated cellular immune activation amongst children who are HEU [7] by showing changes across a suite of soluble markers and demonstrating significant differences in vascular activation.

The drivers of inflammation and immune activation in children who are HEU remain uncertain, but are likely multifactorial. Children who are HEU versus HU have a higher risk of infectious morbidity [16–19], including neonatal sepsis [20]. However, since as CRP, a sensitive marker of sepsis, was not raised in HEU compared with HU children, acute infections are unlikely to explain the differences. Detectable maternal HIV viral load or CMV viremia during pregnancy did not seem to explain all the findings. Children who are HEU had poorer ponderal growth in the first month of life, gaining 6.1 g/day less, or about 180 g less in the first month, than children who are HU. This contrasts with the growth trajectories reported beyond 1 month of age in the same cohort, in which children who are HEU did not display an altered growth trajectory compared to children who are HU [21]. This suggests that any deficit in postnatal growth occurred predominantly during the neonatal period; however, growth was independent of inflammatory biomarker concentrations in our analyses, suggesting another mechanism underlies lower growth in children who are HEU.

This study demonstrates evidence of vascular inflammation amongst HEU children; 4 of 5 endothelial activation markers were significantly higher in HEU compared with HU children (eotaxin, L-selectin, thrombopoietin, and VCAM-1, but not ICAM-1). Endothelial dysfunction has been well described in HIV infection and has been associated with an enhanced risk of cardiovascular disease. For example, VCAM-1 is a marker of vascular injury often raised in HIV infection in adults [22] and children [23] and has been associated with obesity, insulin resistance, and subclinical atherosclerosis. In children living with HIV in Zimbabwe, VCAM-1 has been associated with HIV disease severity (defined by CD4 count and HIV viral load) and abnormal cardiac function [24]. Although the long-term noncommunicable disease risk is not currently known in children who are HEU, a systematic review found several studies reporting elevated triglycerides, oxidative stress and vascular dysfunction, and increased hypertension in 1 small cohort in Spain [25]. The proposed mechanisms of endothelial dysfunction in HIV infection include oxidative stress, dysfunctional regulation of endothelial nitric oxide synthase, and an increased expression of vascular adhesion molecules [26]. Vascular adhesion molecule-1 expression is activated by systemic proinflammatory cytokines, including those that are elevated in this cohort, such as TNF-alpha [27], and HIV-1 tat, a viral protein involved in HIV replication that may directly activate endothelial cells to induce VCAM-1 expression [28], which could plausibly follow fetal exposure to maternal HIV proteins. Alternatively, abnormal endothelial activation markers may indicate neonatal responses to placental dysfunction; the placenta provides an interface between maternal and fetal physiology [29, 30], and because fetal programming may establish the set-point of physiological and metabolic responses that continue throughout life [31, 32], placental dysfunction may drive poor health outcomes in HIV-exposed children. Maternal HIV infection has been linked to proinflammatory placental responses [33], increased trophoblast interferon production [34], and structural abnormalities such as larger placental size and fetal growth restriction [35] and twice the risk of maternal vascular malperfusion [36]. HIV-exposed but uninfected children also showed elevated angiogenesis-related growth factors, including IGFBP-3, GM-CSF, GCSF, angiopoietin-1, and PlGF, which are crucial in tissue growth and repair. However, principal components containing these biomarkers were not associated with increased growth. This may reflect the fact that high levels are responses to pathological processes, rather than being secreted to stimulate growth. Angiogenesis is a tightly regulated activity that is vital during embryonic development, and studies have demonstrated associations between angiogenesis biomarkers and clinical outcomes after preterm birth [37], which may result from a dysregulated inflammatory process [38, 39]. For example, upregulation of placental growth factor (PIGF) is associated with fewer ischemia-induced complications [40] and so may be an attempt to mitigate placental dysfunction during embryology or to repair tissues following a proinflammatory pregnancy. Overall, our findings suggest abnormal endothelial homeostasis in children born to mothers with HIV infection.

This study has several strengths and limitations. This cohort allowed evaluations of multiplex inflammatory biomarkers in a rural African context. Children in this setting were universally breastfed regardless of HIV exposure [41], and all children who were HEU included in this substudy were confirmed to be HIV-free at later timepoints, meaning our results were not related to new, undiagnosed HIV infections. However, these analyses were not the primary aims of the original trial, though we have attempted to control for type 1 error, and laboratory analyses were reliant on sample collection and storage. Bias could have been introduced by selecting children with available samples, and technical factors relating to transportation and long-term storage may have affected laboratory assays, although would be expected to affect HEU and HU samples equally. Additionally, we only used one timepoint to analyze HIV and CMV due to the timing of study visits and sample availability. We did not test for infant CMV before 3 months of age and did not analyze infant antiretroviral therapy (ART) or co-trimoxazole prophylaxis that may have affected results amongst HEU children.

In summary, children who are HEU have significantly elevated systemic and vascular inflammation compared with children who are HU. The first thousand days is a sensitive period of neurodevelopment and organ growth, so future studies should explore associations between inflammation and a range of diverse clinical outcomes, including mortality, development of the immune system, and neurocognitive outcomes. Endothelial activation is particularly pronounced, potentially highlighting vascular inflammation as a distinct inflammatory feature within this population. Given there are currently treatments that could help mitigate long-term negative cardiovascular outcomes, studies should particularly assess future risk of cardiovascular disease in this expanding population.

## Supplementary Material

ofaf810_Supplementary_Data

## Supplementary Data

Supplementary materials are available at *Open Forum Infectious Diseases* online. Consisting of data provided by the authors to benefit the reader, the posted materials are not copyedited and are the sole responsibility of the authors, so questions or comments should be addressed to the corresponding author.
