# Supplementary material for: Systemic Inflammation and Growth in Children Born to Mothers With and Without HIV in Rural Zimbabwe
Source: Open Forum Infect Dis. 2026 Jan 5;13(2):ofaf810. doi: 10.1093/ofid/ofaf810 (PMC12888383; doi:10.1093/ofid/ofaf810)
Supplement: ofaf810_Supplementary_Data [file ofaf810_supplementary_data.docx]

**Supplementary Figure 1. Flow of participants through trial.**

**1** woman enrolled once for two pregnancies in error

**11** women enrolled twice in error

**5280** pregnant women enrolled from 210 randomized clusters^1^

**5270** pregnant women enrolled from 211 randomized clusters^1^

**49** women exited

**90** lost to follow up antenatally

**82** additional fetuses

**252** miscarriages

**113** stillbirths

**4** maternal deaths

**116** fetuses of **114** HIV-unknown mothers

**3989** live infants born to **3937** HIV-negative mothers

**738** live infants born to **726** HIV-positive mothers

**143** matched HIV-unexposed children selected for immunology sub-study

**143** HIV-exposed uninfected children selected for immunology sub-study

**72** children without sufficient sample availability for multiplex assays

**81** children without sufficient sample availability for multiplex assays

**62** HIV-unexposed children included in this analysis

**71** HIV-exposed uninfected children included in this analysis

Appendix Table 1: Biomarker changes in children who are HIV-exposed uninfected compared to children who are HIV-unexposed

^1^Variables adjusted for: age in days, sex, trial arm, birthweight, maternal age, and prematurity. Adjustment for multiple comparisons were done using the Benjamini-Hochberg procedure to control the false discovery rate (FDR). The Adjusted P-value displayed is the calculated q-value.

| **Short name** | **Full name** | **Method** |
| --- | --- | --- |
| CRP | C-reactive protein | ELISA |
| sCD14 | Soluble CD14 | ELISA |
| sCD163 | Soluble CD163 | ELISA |
| LBP | Lipopolysaccharide-binding-protein | ELISA |
| TNFα | Tumour necrosis factor-alpha | Luminex panel |
| IL-6 | Interleukin-6 | Luminex panel |
| IL-33 | Interleukin-33 | Luminex panel |
| IL-8 | Interleukin-8 | Luminex panel |
| L-selectin | L-selectin | Luminex panel |
| IL-10 | Interleukin-10 | Luminex panel |
| EGF | Epidermal growth factor | Luminex panel |
| VEGF | Vascular endothelial growth factor | Luminex panel |
| IL-2 | Interleukin-2 | Luminex panel |
| IL-1β | Interleukin-1β | Luminex panel |
| IFN-γ | Interferon-gamma | Luminex panel |
| IL-1ra | Interleukin -1 receptor antagonist | Luminex panel |
| CCL3 | Chemokine (C-C motif) ligand 3 | Luminex panel |
| CCL4 | Chemokine (C-C motif) ligand 4 | Luminex panel |
| D-dimer | D-dimer | Luminex panel |
| GM-CSF | Granulocyte-macrophage colony-stimulating factor | Luminex panel |
| P-selectin | P-selectin | Luminex panel |
| GCSF | Granulocyte colony-stimulating factor | Luminex panel |
| TPO | Thrombopoietin | Luminex panel |
| VCAM-1 | Vascular cell adhesion molecule 1 | Luminex panel |
| ICAM-1 | Intercellular adhesion molecule 1 | Luminex panel |
| Angiopoietin | Angiopoietin-1 | Luminex panel |
| PlGF | Placental growth factor | Luminex panel |
| Eotaxin | Eotaxin-1 (CCL11) | Luminex panel |
| IGFBP-3 | IGF-binding protein 3 | Luminex panel |

**Supplementary Table 1. List of the biomarkers analysed.**

Luminex kit Assay code: B829YuzuZ; R&D systems, Oxford, UK.

Table 2: Biomarker changes in children who are HIV-exposed uninfected compared to children who are HIV-unexposed

^1^Variables adjusted for: age in days, sex, trial arm, birthweight, maternal age, and prematurity. Adjustment for multiple comparisons were done using the Benjamini-Hochberg procedure to control the false discovery rate (FDR). The Adjusted P-value displayed is the calculated q-value.


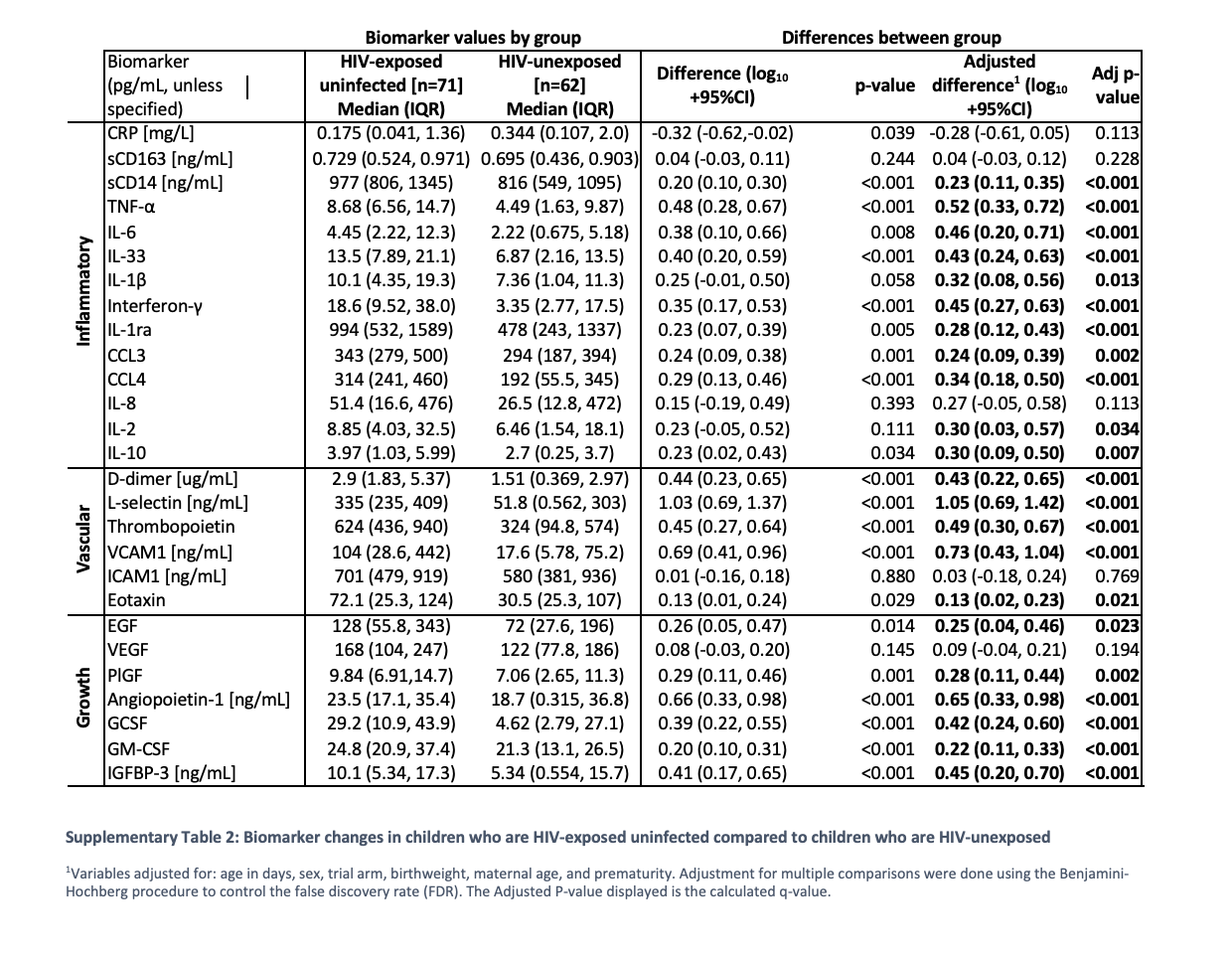


Table 2: Biomarker changes in children who are HIV-exposed uninfected compared to children who are HIV-unexposed

^1^Variables adjusted for: age in days, sex, trial arm, birthweight, maternal age, and prematurity. Adjustment for multiple comparisons were done using the Benjamini-Hochberg procedure to control the false discovery rate (FDR). The Adjusted P-value displayed is the calculated q-value.
